# Supplementary material for: Anisotropic resistance with a 90° twist in a ferromagnetic Weyl semimetal, Co2MnGa
Source: Nat Commun. 2023 Oct 18;14:6583. doi: 10.1038/s41467-023-42222-5 (PMC10584932; doi:10.1038/s41467-023-42222-5)
Supplement: Supplementary file 1 — Supplementary Information [file 41467_2023_42222_MOESM1_ESM.pdf]

Supplemental Information for *Anisotropic resistance with a 90-degree twist in a ferromagnetic Weyl semimetal, Co_2MnGa*

Nicholas P. Quirk[1], Guangming Cheng[2], Kaustuv Manna[3], Claudia Felser[3], Nan Yao[2], and N. P. Ong[1]

[1]*Department of Physics, Princeton University, Princeton, NJ 08544, USA*

[2]*Princeton Institute for the Science and Technology of Materials Princeton University, Princeton, NJ 08544, USA*

[3]*Max Planck Institute for Chemical Physics of Solids, Nöthnitzer Str. 40, 01187 Dresden, Germany*

A. Complete in-plane anisotropy in Sample O1

Table S1 contains the full set of in-plane $R_{ij,kl}$ ($T = 290$ K, $H = 0$ T) for Sample O1 (Fig. 3a of the main text) and compares each measurement to the expected value for the isotropic equivalent. The first 7 rows of each subtable provide the resistances shown in Fig. 3a. The lower 7 rows give the resistances for current directed along the opposite parallel edge on that face, i.e., $ij = 14 \rightarrow ij = 23$ ($\parallel \hat{\mathbf{y}}$), which were omitted from Fig. 3a. They are nearly identical, as expected (Onsager relation at $H = 0$ T). Values in bold font indicate voltage drops that deviate significantly from the isotropic-equivalent value (depicted with red arrows in Fig. 3a.) The 90°-twist anisotropy is apparent in the fact that bold values only appear in the second and third subtables (counted from left to right), corresponding to current directed in the upper (lower) face along $\hat{\mathbf{x}}$ ($\hat{\mathbf{y}}$).

B. Sample N2, $d = 400$ nm

A large 90°-twist resistance anisotropy invariably arises in every thin lamella sample we have studied. Here we discuss in detail the anisotropy observed in a second sample (N2) with a thickness of 400 nm. Tables S2 and S3 give the full $R_{ij,kl}$ for Sample N2 at $T = 290$ K and $H = 0$ T. The anomalous resistances are again presented in bold font. Figure S1 shows the temperature dependences of the four in-plane resistances ($H = 0$ T). All four are qualitatively similar to those of Sample O1 (Fig. 1b in the main text), including the metallic temperature dependences of the high-conductance-axis resistances and the anomalous profiles of the low-conductance-axis resistances. The abrupt jump in $R_{1'4',2'3'}$ at $T \approx 120$ K was not seen in other samples (e.g., O1). We believe it is an artifact due to thermal cycling, not an intrinsic property of the sample. It does not affect the transport results. The magnitude of the anisotropy is somewhat lower (at most about 5×) in this sample and not as symmetric between x and y as in Sample O1. Additionally, the high-conductance-axis resistances ($R_{1'2',4'3'}$ and $R_{14,23}$) do not perfectly match. Furthermore, we note that the isotropic-equivalent value quoted (0.478 Ω, open star in Fig. S1) is the simulated value for a 600 nm thick slab. The closed star gives the isotropic equivalent value for a 400 nm slab: 0.716 Ω. This value increases exponentially with decreasing sample thickness so the deviation may be indicative of some uncertainty in the thickness measurement of the device (SEM imaging).

1. *Issue with lower-face anisotropy in Sample N2*

In this sample (N2), the magnitudes of the low-conductance-axis resistances vary by about 3 times. On the upper face $R_{xx} \simeq 6.5 \times R_{yy}$, but on the lower face $R_{yy} \simeq 1.6 \times R_{xx}$. We believe this imbalance arises from disorder at contact 3' or 4' that impedes the contact from sensing the full anisotropy in the lower surface.

Whereas subtables 1, 2, and 4 (numbered from left to right) in Table S2 for Sample N2 match the symmetry (bolded vs. unbolded) of Table S1 (Sample O1), subtable 3 does not. In order to match the 90°-twist anisotropy seen in Sample O1, $R_{1'4',33'} = 0.05\ \Omega$ and $R_{2'3',44'} = 0.05\ \Omega$ should be near the 1-Ω level. Every other in-plane $R_{ij,kl}$ matches the expectation for the 90°-twist anisotropy. ($R_{1'4',22'}$ and $R_{2'3',11'}$ are a little lower, ∼0.5 Ω rather than 1 Ω, but they are still much larger than the isotropic expectation.) Due to the fact that the four-point resistances $R_{1'4',33'}$ and $R_{2'3',44'}$ are symmetrically equivalent, the contact issue could be either at the 3' or the 4' corner. This corner issue also can be seen in the z-axis current configurations depicted in Table S3. Whereas subtables 1 and 2 each have four anomalously large resistances (bolded), subtables 3 and 4—with current drain contacts at 3' and 4'—each only have two. $R_{33',44'}$, $R_{33',1'4'}$, $R_{44',33'}$, and $R_{44',2'3'}$ have near-zero resistances but are expected to be about 1 Ω by roto-inversion symmetry. The first two of these configurations share a voltage measurement at 4' and the second two a measurement at 3'. Although the contact works (never exhibit an open-circuit resistance), it is not capable of fully sensing the surface anisotropy. Instead, it only senses the low resistance of the bulk states. The high-conductance-axis measurements using this contact (subtable 4 of Table S2), which probe the bulk states, appear normal. We suspect that disorder was induced at one of these corners while mounting this very thin lamella onto the substrate. The surface anisotropy then avoids this area and the contact does not properly sense an anisotropy. It is possible that the reduced dimensionality of the anisotropic surfaces states may make them especially sensitive to surface disorder. Regardless of this issue, however, this sample still clearly exhibits a 90°-twist anisotropy. It is pristine on the upper face but improperly sensed by this "bad contact" on the lower face.

C. Thickness, aspect ratio, and lattice orientation studies

In addition to Samples O1 and N2, we fabricated four other thin-plate crystals with contacts on both the upper and lower faces (Fig. S2). Their geometrical details and resistance anisotropies are listed in Table S4.

1. *Thickness dependence*

The first four samples listed in Table S4 all have the same lateral geometry but different thicknesses, d. In the Van der Pauw corner-contact geometry as d is increased, the expected value of each in-plane resistance in the isotropic equivalent sharply decreases. The expected isotropic values are 0.478, 0.287, 0.095, and 0.058 Ω for samples with $d = 0.6$ (N2), 1 (O1), 3 (D1), and 5 µm (M1), respectively. In each sample, the measured high-conductance-axis resistances ($R_{14,23}$ and $R_{1'2',4'3'}$) are close to these expected isotropic values and the low-conductance-axis resistances are much larger, by 2× (N2) - 58× (M1). This observation

agrees with the "thin-surface" model described in the main text. If the anisotropic conductivity is limited to states in the upper and lower surfaces, then the low-conductance-axis resistances should not change significantly with sample thickness.

2. Lattice orientation dependence

In order to determine if the twisted anisotropy depends strongly on the alignment of the lamella to the crystal lattice, we made a sample with edges defined at a 45° in-plane angle with respect to a facet of the source crystal (Sample F1*). This device still exhibits a robust 90°-twist resistance anisotropy. The high-conductance-axis resistances agree with the isotropic-equivalent expectation (0.239 Ω): $R_{14,23} = 0.211$ Ω and $R_{1'2',4'3'} = 0.250$ Ω and the low-conductance-axis resistances are about 2-5 times greater: $R_{12,43} = 0.77$ Ω and $R_{1'4',2'3'}$ = 0.473 Ω.

3. Aspect ratio dependence

In order to determine if the square shape of the devices is essential to realizing the 90°-twist anisotropy, we fabricated a sample with a 2:1 aspect ratio (J1) (14 × 7 × 1 µm). Due to the Van der Pauw geometry, the rectangular aspect ratio generates a large difference between the resistance measured along the long side $\vec{ij} \parallel \hat{\mathbf{x}}$ and the short side $\vec{ij} \parallel \hat{\mathbf{y}}$. The expected isotropic-equivalent resistances in this geometry are 1.457 and 0.012 Ω for $\vec{ij} \parallel \hat{\mathbf{x}}$ and $\vec{ij} \parallel \hat{\mathbf{y}}$, respectively. On the upper face this geometry-induced anisotropy aligns with the high-conductance axis ($\hat{\mathbf{y}}$). On the lower face it counteracts the intrinsic anisotropy ($\parallel \hat{\mathbf{x}}$). In this rectangular sample, we measure $R_{14,23} = 0.016$ Ω, which matches the isotropic equivalent. However, we measure $R_{12,43} = 4.442$ Ω, which is anomalously large. On the lower face, the two resistances are roughly equal in magnitude, but due to the geometric effect, $R_{1'4',2'3'}$ = 1.58 Ω is anomalously large and $R_{1'2',4'3'} = 1.269$ Ω agrees with the isotropic-equivalent expectation. Thus, the 90°-twist anisotropy arises similarly in this 2:1 rectangular sample as in a square one.

D. Additional weak magnetoresistances

At low field ($H < 1.3$ T), each $R_{ij,kl}$ exhibits weak magnetoresistance (MR) that is anisotropic and, in some configurations, field-antisymmetric. At high-field ($H > 1.3$ T, measured up to 9 T), each MR is small, negative, and featureless; each resistance decreases by ∼20 mΩ at $H = 9$ T. Figure S3a depicts the MR of the four in-plane $R_{ij,kl}$ in Sample O1 at $T = 50$ K ($\mathbf{H} \parallel \hat{\mathbf{z}}$). This plot is qualitatively identical to Fig. 3b of the main text (for Sample N2). As in Sample N2, below a magnetization-saturation field $H_S = 1.3$ T, the MR measured with current $\parallel \hat{\mathbf{y}}$, $R_{14,23}$ and $R_{1'4',2'3'}$, have local maxima at $|H| \simeq 0.75$ T and the MR $\parallel \hat{\mathbf{x}}$, $R_{12,43}$ and $R_{1'2',4'3'}$, have minima. The MR $\parallel \hat{\mathbf{y}}$ have a significant H-antisymmetric component; the MR $\parallel \hat{\mathbf{x}}$ do not. Figure S3b shows the field-antisymmetrization of the MR $\parallel \hat{\mathbf{y}}$ on the same plot as the Hall resistances. They have a similar profile to the anomalous Hall curves and share the same knee at $|H| \simeq 1.3$ T. However, they are less than the Hall resistance by a factor of 10.

1. *Field-antisymmetric in-plane magnetoresistances*

We find additional antisymmetric magnetoresistances when the magnetic field is directed in the x-y plane (Fig. S4). These H-antisymmetric in-plane MR present an additional, interesting symmetry in these thin crystals.

We start with the in-plane MR ($\parallel \hat{\mathbf{x}}$, $\hat{\mathbf{y}}$) measured on the left narrow side face of the crystal, $R_{14,1'4'}(H)$. This faces lies in the $\hat{\mathbf{y}} - \hat{\mathbf{z}}$ plane. Figure S4a shows that both the MR $\parallel \hat{\mathbf{x}}$ and $\parallel \hat{\mathbf{y}}$ have a low-field H-antisymmetric component with a negative slope. Figure S4e plots the H-antisymmetrization of each in-plane $R_{ij,i'j'}(H)$. The blue curves correspond to $R_{14,1'4'}(H)$. The magnitude of the H-antisymmetric component in this face is about twice as large for $\mathbf{H} \parallel \hat{\mathbf{y}}$ than for $\mathbf{H} \parallel \hat{\mathbf{x}}$, i.e., it is larger when H is *aligned with* the applied-current axis ($\hat{\mathbf{y}}$). On the right face, $R_{23,2'3'}$ (also in $\hat{\mathbf{y}} - \hat{\mathbf{z}}$) has an H-antisymmetric component with the same magnitude and directional dependence, but opposite sign. (See Fig. S4b and the red curves in Fig. S4e.) The low-field, in-plane MR of the left and right faces are mirror images of each other.

Now we examine the in-plane MR of the front and back side faces, $R_{12,1'2'}$ and $R_{43,4'3'}$ ($\hat{\mathbf{x}} - \hat{\mathbf{z}}$ planes). When $\mathbf{H}$ is parallel to the applied-current axis ($\hat{\mathbf{x}}$), the MR have H-antisymmetric components equal in magnitude to those of the left and right faces for $\mathbf{H}$ in the same scenario ($\mathbf{H} \parallel \hat{\mathbf{y}}$). These are depicted with solid lines in Fig. S4c (Fig. S4d) for $R_{12,1'2'}$ ($R_{43,4'3'}$). Again, the signs of the slopes mirror each other. The sign of the H-antisymmetric slope of $R_{12,1'2'}$ matches that of $R_{14,1'4'}$ (+), and $R_{43,4'3'}$ matches $R_{23,2'3'}$ (-) (Fig. S4e). Perhaps this suggests that the same mechanism generates the antisymmetric MR in the left and back faces (sharing the $11'$ edge) and likewise in the front and right faces (sharing the $33'$ edge). Finally, as opposed to the left/right faces, the front/back faces feature entirely symmetric MR when the current is perpendicular to the field, e.g., $R_{43,4'3'}(\mathbf{H} \parallel \hat{\mathbf{y}})$ (dotted pink curves in Figs. S4d, e). Note that this orientation could not be measured on the front side, $R_{12,1'2'}$, because a contact was broken by an operational error, ending measurements on this device. (There is no dotted curve in Fig. S4c.)

In summary, the in-plane antisymmetric MR measured in the narrow side faces ($\hat{\mathbf{y}} - \hat{\mathbf{z}}$ and $\hat{\mathbf{x}} - \hat{\mathbf{z}}$ planes) of these thin crystals present an additional interesting magnetotransport pattern with a different symmetry than the intrinsic 90°-twist anisotropy. The mechanism that generates this symmetry is unknown to us. We note that these in-plane MR are very small ($\sim$mΩ) in comparison to the underlying 90°-twist anisotropy (Ω) and represent only a small perturbation to the otherwise field-independent $R_{ij,kl}$.

E. Extended symmetry analysis

We consider the configuration $R_{11',aa'}$ (see diagram in Fig. S5). For simplicity, we take the crystal symmetry to be $C_{4z}M_z$ (as $M_z = IC_{2z}$, this is equivalent to $C_{4z}I$ plus the 2-fold rotation C_{2z}). The applied current density leads to a voltage contour pattern that transforms as a subgroup G_c of the full point group $\mathcal{G}$ of the twisted lattice ($G_c \subset \mathcal{G}$). The observed potential contours are invariant (up to an overall sign) under any operation g belonging to the subgroup ($g \in G_c$). For example, a reflection M_z across the x-y midplane followed by a dihedral reflection M_d across the diagonal plane normal to $\hat{\mathbf{n}} = (\hat{\mathbf{x}} + \hat{\mathbf{y}})/\sqrt{2}$ leaves the contours invariant apart from a sign change. Hence $M_d M_z \in G_c$. Likewise, the rotation C_{2d} by π about a diagonal in the x-y mid-plane also belongs to G_c.

We focus on the two side faces 233'2' and 344'3' and adopt the "flattened" representation with planar coordinates (u, z) and origin at the midpoint of 33' (see Fig. S5). The potential is defined to be zero at the origin. Both the symmetry operations $M_d M_z$ and C_{2d} correspond to rotation by π about the axis normal to the (u, z) plane, which leads to $u \to -u$ and $z \to -z$. Acting alone, M_d inverts the sign of u while M_z inverts the sign of z.

Under any operation $g \in G_c$, $V(u, z)$ transforms as

$$\mathcal{P}_g V(\mathbf{u}) = V(g^{-1}\mathbf{u}), \tag{1}$$

where $\mathbf{u} = (u, z)$. For $g = C_{2d}$ $((u, z) \to (-u, -z))$, the potential contours remain invariant but acquire a sign change. Hence $V(u, z)$ satisfies

$$V(u, z) = -V(-u, -z). \tag{2}$$

This is our first constraint. A second constraint is that $V(u, z)$ must not remain invariant under the reflection $u \to -u$ (effected by M_d alone),

$$\mathcal{P}_{M_d} V(u, z) = V(-u, z) \neq V(u, z). \tag{3}$$

This is because the edges 32 and $3'4'$ are identical twins as are the edges 3'2' and 34, but the two pairs are distinct.

Together, Eqs. 2 and 3 suggest that, in the flattened representation, z appears in $V(u, z)$ in the combination $z.\text{sign}(u)$. The potential contours that satisfy Eqs. 2 and 3 then have the form

$$V(u, z) = U(u)F(z.\text{sign}(u) - c), \quad (c \neq 0) \tag{4}$$

where U is any function that is odd in its argument $[U(-u) = -U(u)]$ and F is an arbitrary function of $z.\text{sign}(u) - c$, with c a non-zero constant. Note that if $c = 0$, $V(u, z)$ violates constraint Eq. 3. Similarly, a surface E-field that is uniform ($V \sim \mathbf{a} \cdot \mathbf{u}$ with any constant $\mathbf{a}$) trivially satisfies Eq. 2 but is excluded by Eq. 3.

The simplest example of Eq. 4 is

$$V(u, z) = u(z.\text{sign}(u) - c), \quad (c \neq 0). \tag{5}$$

Fixing $c = d/2$, Eq. 5 displays the symmetry observed for $V(u, z)$ in our experiment. Specifically, both Eqs. 4 and 5 predict that $V = 0$ all along the edge 33' $(u = 0)$. Moreover, $V = 0$ along the edges 32 and 3'4' with the choice $c = d/2$. Consequently, the zero-potential edges are strictly enforced by the 90°-twist geometry.

1. *Negative non-local resistance*

Although the above symmetry arguments enforce $V = 0$ along edges 32, 3'4', and 33', when current is directed along ij = 11', in the experiment these edges have very small finite values (Fig. 2b). In particular, the non-local resistance along the diametrically opposed edge, e.g., $R_{11',33'}$, always has a tiny negative value. (The voltage drop is anti-parallel to the applied-current direction.) The simulated models also capture this negativity, as shown in the column charts of Figs. 4b and 5b. These small negative values appear to arise from

isotropic contributions from bulk states that are not directly considered in this symmetry analysis.

F. Alternate scenarios to the anisotropic surface-states picture

We converged to the model based on anisotropic surface states after exploring several alternative scenarios. Each of these scenarios was abandoned after we found that it conflicted with observations or its predictions disagreed with subsequent measurements. We summarize some of these in this section.

1. Highly conducting domain walls

In magnetic Weyl semimetals, theory predicts that the domain walls separating magnetic domains feature chiral states and are highly conducting even when buried in the bulk of the crystal. Hence it is plausible that they could be the source of the observed anisotropies. However, despite investigating a range of domain-wall configurations (satisfying the C_4I symmetry), we did not find one that could account for the entire set of non-local resistances $R_{ij,kl}$. In particular, the 1-Ohm resistance observed with current applied at one vertical edge (e.g. $\mathrm{R}_{11'44'}$) could not be reproduced. Eventually, the strongest argument against this approach was the empirical finding that when we align all domains in an external magnetic field H, $R_{ij,kl}$ is barely affected (even up to 9 T). Direct evidence for the alignment of all domains at low field comes from the anomalous Hall effect shown in Figs. 3d-f. This persuaded us that the unusual electrical anisotropies are intrinsic features of the twisted lattice and are unrelated to domain walls.

2. Resistance network (edge conduction) model

In a second approach, we assumed that the applied current flows strictly along the twelve edges of the crystal (no current enters the bulk). This approach was motivated by theories of higher-order topological insulators. The sample can be represented by a network of twelve resistors. The C_4I symmetry reduced the number of distinct resistance values. The symmetry constraints allowed a subset of the $R_{ij,kl}$ to be simulated. However, none of the trials accounted for the entire set. Eventually, we abandoned this approach because we found that the assumption of zero bulk current was untenable. The set of $R_{ij,kl}$ values segregate into two groups, with high and low values. After we performed numerical calculations based on matching $R_{ij,kl}$ to the isotropic equivalent, we found that the isotropic-equivalent calculations successfully accounted for all the values in the low-resistance subgroup, implying that current actually flows throughout the bulk. The anomalous findings arise from the unusually large values observed in certain configurations.

3. Twisted anisotropic bulk states

The simplest picture we could imagine that might generate a planar anisotropy with a 90° twist was a union of two anisotropically conducting slabs, one on top of the other, with

high conductance along the y axis in the upper slab and along the x axis in the lower. This "two-slab" model was the starting point of our electrostatic simulations. Although the abrupt planar boundary between the two slabs seemed unphysical to us, attempts to simulate a planar anisotropy that gradually twists along z (many thin segments with incrementally rotated planar conductivity tensors) quickly became computationally untenable. Surprisingly, we found that this extremely simple two-slab simulation was able to generate a planar anisotropy with a 90° twist. (In Fig. 5, δ = 500 nm is the two-slab model). However, as mentioned in the main text, no matter what α and β were chosen we could not generate an anisotropy that agreed with all the experimental $R_{ij,kl}$. We then adopted a "3-slab" picture, adding the central isotropic section. This required a huge suppression of the z-axis conductivity in the thin surfaces ($\beta \approx 5.76 \times 10^4$ for $\delta = 10$ nm) in order for them to not be shorted out by the isotropically conducting bulk. We found that as we decreased the thickness of the anisotropic surface sections (Fig. 5b) the anomalous z-axis resistances systematically approached better agreement with the experiment ($\sim 1\ \Omega$). This rather strange picture comes closer than any other model to matching the experimentally observed anisotropy in these thin crystals. There are some important outstanding questions, however, such as: why does the thin-surface simulation fail for the resistances of the side faces (e.g., $\mathrm{R}_{12,1'2'}$)? It undershoots these by roughly 50% of the experimental values (which match the isotropic equivalent expectation). In order to make the computations feasible, we did not consider the possibility of additional surface states on the narrow side faces of the sample. Adding this complexity could be an powerful next step to fully unraveling the peculiar twisted anisotropy in these samples.

i,j	k,l	Iso. Equiv. R (Ω)	Sample O1 R (Ω)	i,j	k,l	Iso. Equiv. R (Ω)	Sample O1 R (Ω)	i,j	k,l	Iso. Equiv. R (Ω)	Sample O1 R (Ω)	i,j	k,l	Iso. Equiv. R (Ω)	Sample O1 R (Ω)
1,4	2,3	0.29	0.2	1,2	4,3	0.29	**1.9**	1’,4’	2’,3’	0.29	**2.5**	1’,2’	4’3’	0.29	0.18
	2,2’	-10^{-8}	-0.03		4,4’	10^{-8}	**1.0**		2,2’	-10^{-8}	**-1.5**		4,4’	10^{-8}	0.04
	3,3’	10^{-8}	0.04		3,3’	-10^{-8}	**-0.7**		3,3’	10^{-8}	**1.0**		3,3’	-10^{-8}	-0.05
	1’,2’	1.87	2.1		1’,2’	4.0	4.4		1,2	1.87	2.1		1,2	4.0	4.6
	2’,3’	0.29	0.3		2’,3’	-1.87	-2.0		2,3	0.29	0.3		2,3	-1.87	-2.1
	4’,3’	-1.87	-1.3		4’,3’	0.29	0.2		4,3	-1.87	-1.2		4,3	0.29	0.25
	1’,4’	4.0	3.7		1’,4’	1.87	2.0		1,4	4.0	3.8		1,4	1.87	2.0
2,3	1,4	0.29	0.2	4,3	1,2	0.29	**1.9**	2’,3’	1’,4’	0.29	**2.7**	4’,3’	1’,2’	0.29	0.15
	1,1’	-10^{-8}	-0.06		1,1’	10^{-8}	**1.0**		1,1’	-10^{-8}	**-1.5**		1,1’	10^{-8}	0.02
	4,4’	10^{-8}	0.07		2,2’	-10^{-8}	**-0.7**		4,4’	10^{-8}	**1.0**		2,2’	-10^{-8}	-0.05
	1’,2’	-1.87	-2.1		1’,2’	0.29	0.3		1’,2’	-1.87	-2.0		1,2	0.29	0.2
	2’,3’	4.0	4.1		2’,3’	1.87	1.6		2’,3’	4.0	4.1		2,3	1.87	1.6
	4’,3’	1.87	1.6		4’,3’	4.0	3.1		4’,3’	1.87	1.7		4,3	4.0	3.1
	1’,4’	0.29	0.4		1’,4’	-1.87	-1.2		1’,4’	0.29	0.3		1,4	-1.87	-1.3

TABLE S1. **Sample O1 in-plane resistances.** (T = 290 K, H = 0 T.) The first two columns identify $R_{ij,kl}$ according to the convention shown in the inset of Fig. S1. The third column shows the simulated resistances for the isotropic equivalent. The last column provides the experimental data. The first (second) subtable is with current directed on the upper face $\parallel \hat{\mathbf{y}}$ ($\parallel \hat{\mathbf{x}}$). The third and fourth subtables correspond to the same current directions on the lower face. Only the values in bold font deviate significantly from the isotropic equivalent.

i,j	k,l	Iso. Equiv. R (Ω)	Sample N2 R (Ω)
1,4	2,3	0.48	0.44
	2,2’	-10^{-7}	-0.05
	3,3’	10^{-7}	0.02
	1’,2’	3.83	2.94
	2’,3’	0.48	0.51
	4’,3’	-3.83	-3.69
	1’,4’	8.15	7.13
2,3	1,4	0.48	0.44
	1,1’	-10^{-7}	-0.05
	4,4’	10^{-7}	0.02
	1’,2’	-3.83	-3.31
	2’,3’	8.15	7.66
	4’,3’	3.83	3.84
	1’,4’	0.48	0.51

i,j	k,l	Iso. Equiv. R (Ω)	Sample N2 R (Ω)
1,2	4,3	0.48	**2.88**
	4,4’	10^{-7}	**1.11**
	3,3’	-10^{-7}	**-0.89**
	1’,2’	8.15	7.07
	2’,3’	-3.83	-3.29
	4’,3’	0.48	0.89
	1’,4’	3.83	2.90
4,3	1,2	0.48	**2.88**
	1,1’	10^{-7}	**1.14**
	2,2’	-10^{-7}	**-0.91**
	1’,2’	0.48	0.83
	2’,3’	3.83	3.86
	4’,3’	8.15	8.41
	1’,4’	-3.83	-3.72

i,j	k,l	Iso. Equiv. R (Ω)	Sample N2 R (Ω)
1’,4’	2’,3’	0.48	**1.07**
	2,2’	-10^{-7}	**-0.51**
	3,3’	10^{-7}	0.05
	1,2	3.83	2.92
	2,3	0.48	0.51
	4,3	-3.83	-3.71
	1,4	8.15	7.16
2’,3’	1’,4’	0.48	**1.07**
	1,1’	-10^{-7}	**-0.51**
	4,4’	10^{-7}	0.05
	1,2	-3.83	-3.27
	2,3	8.15	7.66
	4,3	3.83	3.88
	1,4	0.48	0.51

i,j	k,l	Iso. Equiv. R (Ω)	Sample N2 R (Ω)
1’,2’	4’,3’	0.48	0.69
	4,4’	10^{-7}	0.07
	3,3’	-10^{-7}	-0.07
	1,2	8.15	7.05
	2,3	-3.83	-3.28
	4,3	0.48	0.83
	1,4	3.83	2.95
4’,3’	1,2	0.48	0.69
	1,1’	10^{-7}	0.11
	2,2’	-10^{-7}	-0.09
	1,2	0.48	0.89
	2,3	3.83	3.85
	4,3	8.15	8.41
	1,4	-3.83	-3.67

TABLE S2. **Sample N2 in-plane resistances.** (T = 290 K, H = 0 T.) These tables compare the measured resistances in Sample N2 to the isotropic-equivalent expected values. The bold values are the anomalous $R_{ij,kl}$. Note that the third subtable only has 4 anomalous resistances: $R_{1'4',33'}$ and $R_{2'3',44'}$ do not capture the full anisotropy. We believe this issue to be caused by disorder at contact 3’ or 4’ on the lower face of this thin crystal (see section B1). The sample thickness used in the isotropic equivalent simulation is 600 nm.

ij,kl	Sample N2 R (Ω)
11’,22’	**0.46**
11’,33’	-0.001
11’,44’	**1.03**
11’,23	-0.05
11’,43	**1.14**
11’,2’3’	**-0.51**
11’,4’3’	0.11

ij,kl	Sample N2 R (Ω)
22’,33’	**0.82**
22’,44’	-0.001
22’,11’	**0.46**
22’,14	-0.05
22’,43	**-0.91**
22’,1’4’	**-0.51**
22’,4’3’	-0.09

ij,kl	Sample N2 R (Ω)
33’,44’	0.03
33’,11’	-0.001
33’,22’	**0.82**
33’,14	0.02
33’,12	**-0.89**
33’,1’4’	0.05
33’,1’2’	-0.07

ij,kl	Sample N2 R (Ω)
44’,11’	**1.03**
44’,22’	-0.001
44’,33’	0.03
44’,23	0.02
44’,12	**1.11**
44’,2’3’	0.05
44’,1’2’	0.07

TABLE S3. **Sample N2 out-of-plane resistances.** ($T = 290$ K, $H = 0$ T.) These tables show the resistances measured with current applied $\parallel \hat{\mathbf{z}}$. In the isotropic equivalent each resistance is $< 10^{-6}$ Ω. As with Sample O1, these z-axis current configurations feature anomalous $\sim$ 1-Ω voltage drops and an alternating pattern of high and low resistances that is enforced by the $C_4 I$ symmetry of the 90°-twist anisotropy. The aforementioned issue at contact 3’ (or 4’) affects these measurements as well: the third and fourth subtables each only have two anomalous resistances whereas the first and second each have four.

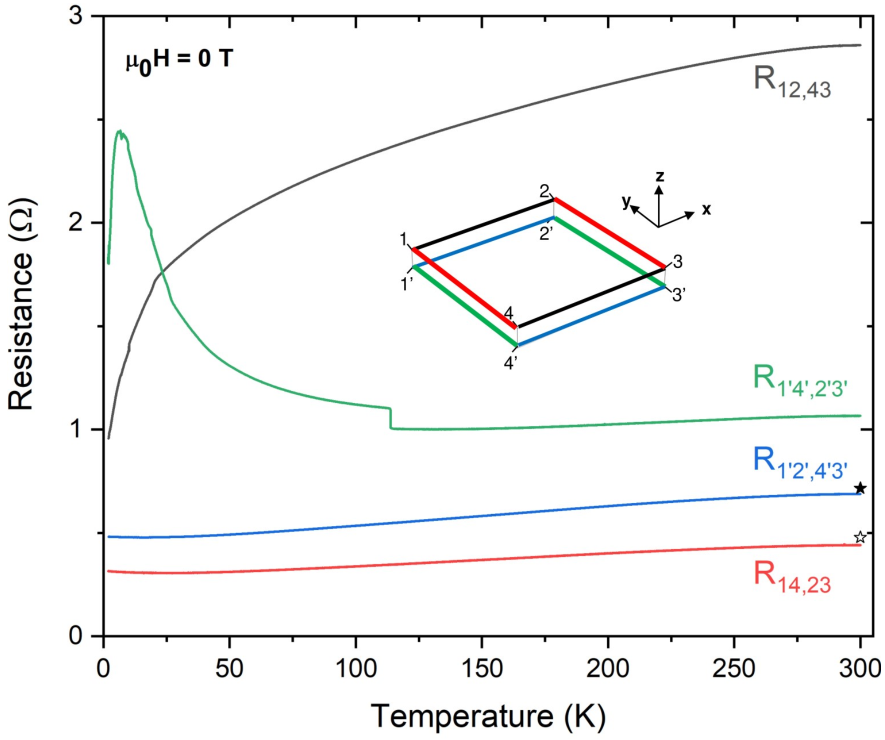

FIG. S1. **Sample N2 temperature dependences.** The low-conductance-axis resistances ($R_{14,23}$ and $R_{1'2',4'3'}$) have metallic profiles matching the isotropic equivalent whereas the high-conductance-axis resistances ($R_{12,43}$ and $R_{1'4',2'3'}$) have anomalous profiles, similar to Sample O1 of the main text. We believe the abrupt jump at $T = 120$ K in $R_{1'4',2'3'}$ to be an artifact of thermal cycling and not an intrinsic feature. The white (black) star symbols at $T = 290$ K give the isotropic equivalent values for $d = 600$ (400) nm.

Samp.	Dimensions (μm)	Iso. Equiv. R (Ω)	$R_{14,23}$ (Ω)	$R_{12,43}$ (Ω)	$R_{1'4',2'3'}$ (Ω)	$R_{1'2',4'3'}$ (Ω)
N2	12 × 12 × 0.4	0.478	0.44	2.88	1.068	0.689
O1	10 × 10 × 1	0.287	0.2	1.9	2.6	0.17
D1	12 × 12 × 3	0.095	0.105	0.928	4.41	0.38
M1	10 × 10 × 5	0.058	0.061	3.355	3.243	0.042
F1*	10 × 10 × 1.2	0.239	0.211	0.77	0.473	0.250
J1	14 × 7 × 1	1.457 ($\parallel \hat{\mathbf{x}}$)<br>0.012 ($\parallel \hat{\mathbf{y}}$)	0.016	4.442	1.58	1.269

TABLE S4. **Sample details.** The first four samples have roughly the same lateral dimensions but varying thicknesses. The fifth (F1*) has edges rotated from alignment with the crystal lattice by an in-plane angle of 45 degrees. The sixth (J1) has a 2:1 rectangular aspect ratio.

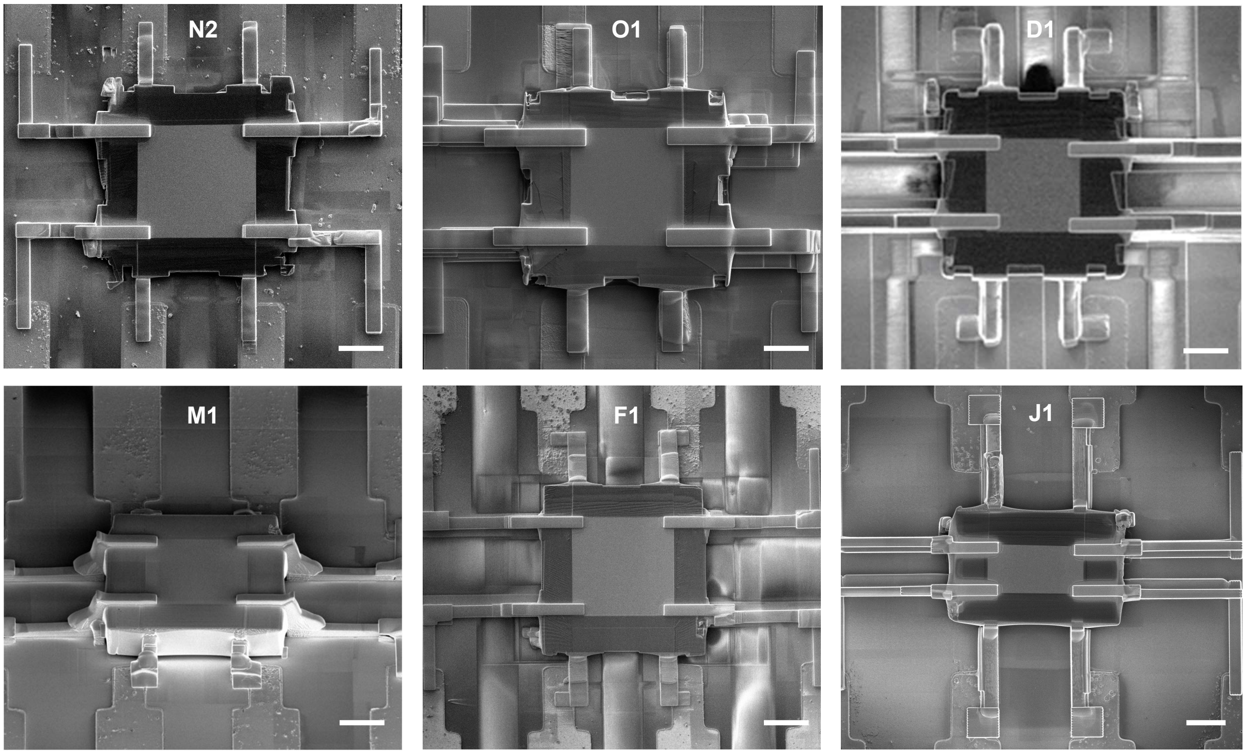

FIG. S2. **SEM micrographs of the thin lamella devices.** Scale bars = 5 μm.

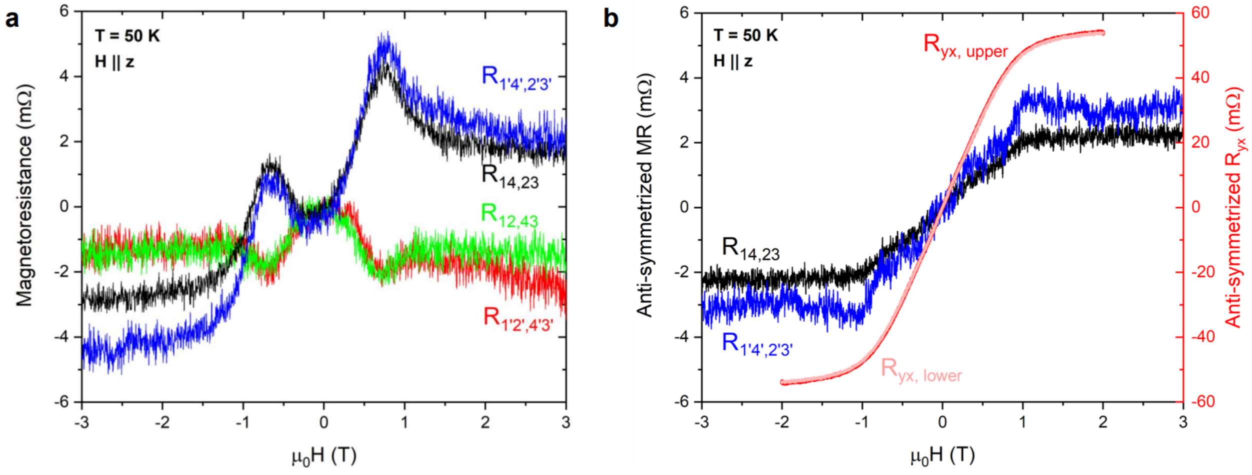

FIG. S3. **Sample O1 magnetotransport** ($\mathbf{H} \parallel \hat{\mathbf{z}}$.) **a.** Magnetoresistances $R(H) - R(H = 0)$ of the four in-plane $R_{ij,kl}$. The MR $\parallel \hat{\mathbf{y}}$ feature a field-antisymmetric component resembling the anomalous Hall resistance, but the MR $\parallel \hat{\mathbf{x}}$ do not. The zero-field values which have been subtracted off reflect the intrinsic 90°-twist anisotropy. At $H = 0$ T ($T = 50$ K), $R_{14,23} = 0.141\ \Omega$, $R_{12,43} = 1.241\ \Omega$, $R_{1'4',2'3'} = 1.61\ \Omega$, and $R_{1'2',4'3'} = 0.114\ \Omega$. **b**. This panel shows the field-antisymmetrized MR $\parallel \hat{\mathbf{y}}$ alongside the anomalous Hall resistances (upper face = dark red, lower face = light red). The two sets of curves share a knee at $|H| \approx 1.3$ T but the anomalous Hall resistances are an order of magnitude larger.

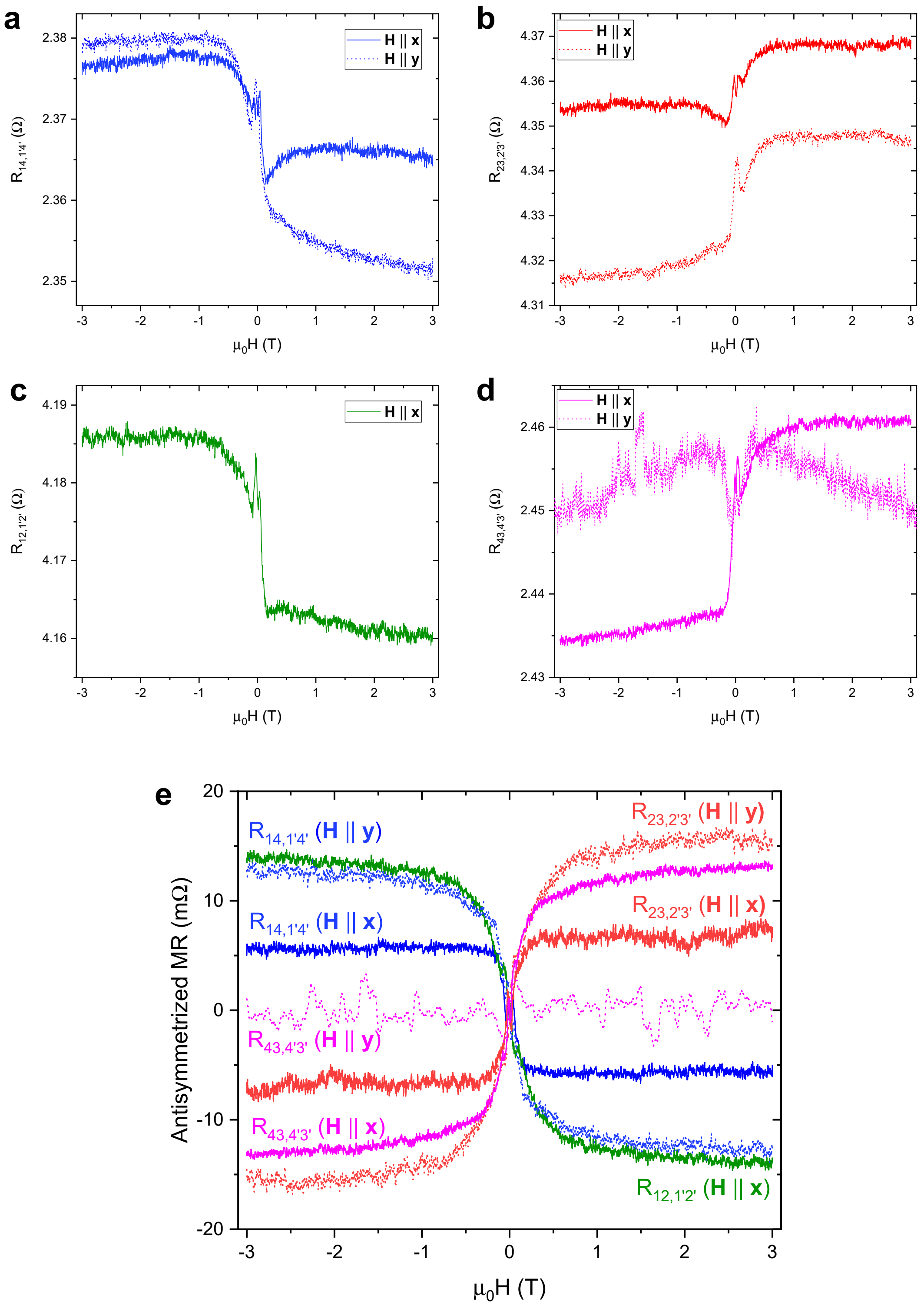

FIG. S4. **In-plane antisymmetric MR. a-d.** These panels show the raw resistances of the side faces ($R_{ij,i'j'}$) with **H** directed in the x-y plane (Sample O1, 50 K). The solid (dashed) curves depict $\mathbf{H} \parallel \hat{\mathbf{x}}$ ($\mathbf{H} \parallel \hat{\mathbf{y}}$). $R_{12,1'2'}$ ($\mathbf{H} \parallel \hat{\mathbf{y}}$) was unable to be measured after a contact broke. **e.** Panels a-d after field antisymmetrization. Interestingly, $R_{14,1'4'}(\mathbf{H} \parallel \hat{\mathbf{y}})$ (left face) is identical to $R_{43,4'3'}(\mathbf{H} \parallel \hat{\mathbf{x}})$ (front). $R_{23,2'3'}(\mathbf{H} \parallel \hat{\mathbf{y}})$ (right) and $R_{12,1'2'}(\mathbf{H} \parallel \hat{\mathbf{x}})$ (back) are identical to each other and are mirror images of the left/front pair.

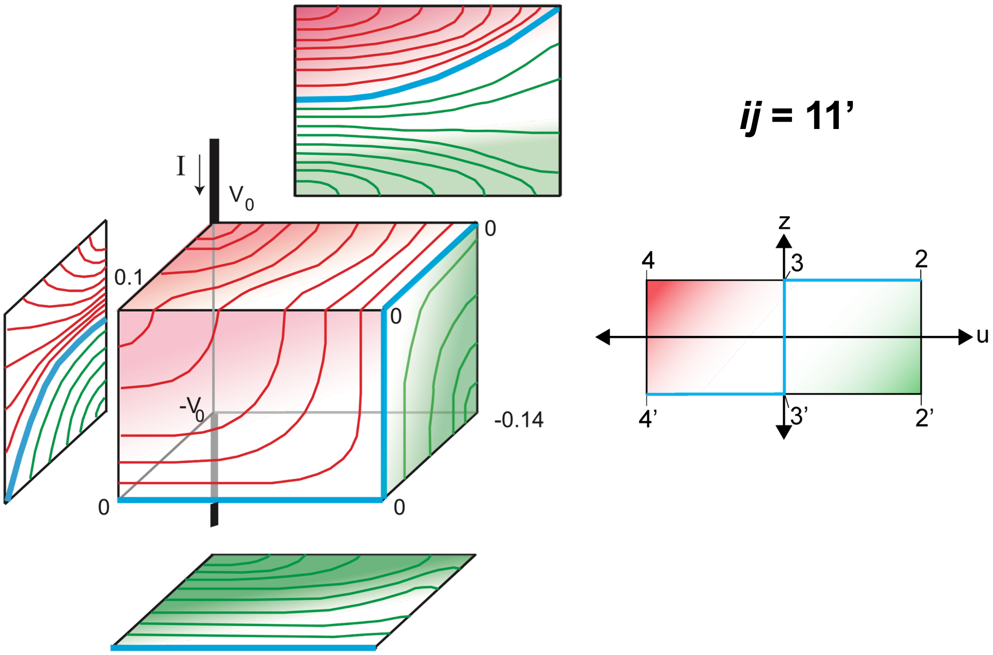

FIG. S5. **Surface potential distribution for I ∥ ẑ.** This diagram shows how the C_4I symmetry gives rise to a surface potential that is zero along the 4’-3’-3-2 edge for current contacts ij= 11’. The “flattened representation” for the analysis in Section E is shown on the right.
